# Supplementary material for: Relief of autoinhibition by conformational switch explains enzyme activation by a catalytically dead paralog
Source: eLife. 2016 Dec 15;5:e20198. doi: 10.7554/eLife.20198 (PMC5201418; doi:10.7554/eLife.20198)
Supplement: Supplementary file 1. — DOI: http://dx.doi.org/10.7554/eLife.20198.021 [file elife-20198-supp1.docx]

**Supplemental File 1. Primers used in molecular cloning of the expression constructs**

| # | ORF to amplify (direction and target construct) | Sequence |
| --- | --- | --- |
| p1 | Prozyme (forward cloning for heterodimer) | 5'‑**AAGGAGATATACATA**TGTCGGTCACGCGGATTAAC‑3' ^a,b^ |
| p2 | Prozyme (reverse cloning for heterodimer) | 5'‑**CTTTACCAGACTCGA**TCAGGCACTGCGTGCGT‑3' ^a,b^ |
| p3 | His_6_-Smt3-*Tb*AdoMetDC (forward cloning for heterodimer) | 5'‑**AGGAGATATACCATG**GGCAGCAG‑3' ^a,b^ |
| p4 | His_6_-Smt3-*Tb*AdoMetDC (reverse cloning for heterodimer) | 5'‑**ATGCGGCCGCAAGCT**TTATTCTTTCGCGCCGCTC‑3' ^a,b^ |
| p5 | *Tb*AdoMetDC (forward subcloning into pCR-Blunt II-TOPO) | 5'‑*TCTAGA*ATGAGCAGCTGCAAAGATAGCC‑3' ^a,c^ |
| p6 | *Tb*AdoMetDC (reverse subcloning into pCR-Blunt II-TOPO) | 5'‑*GGATCC*TTATTCTTTCGCGCCGCTC‑3' ^a,c^ |
| p7 | *Tb*AdoMetDC-H172A (forward mutagenesis) | 5'‑CGATTGATAGCGATgcTTATTTTCTG‑3' ^d^ |
| p8 | *Tb*AdoMetDC-H172A (reverse mutagenesis) | 5'‑CAGAAAATAAgcATCGCTATCAATCG‑3' ^d^ |
| p9 | *Tb*AdoMetDC-H172A (forward cloning for heterodimer) | 5'‑AGAT*GGATCC*ATGAGCAGCTGCAAAGATAGCC‑3' ^a,c^ |
| p10 | *Tb*AdoMetDCΔ26 (reverse cloning for monomer) | 5'‑AGAT*CTCGAG*TTATTCTTTCGCGCCGCTC‑3' ^a,c^ |
| p11 | *Tb*AdoMetDC mutants: Δ26 and H172A (reverse cloning for heterodimer) | 5'‑ AGAT*AAGCTT*TATTCTTTCGCGCCGCTC‑3' ^a,c^ |
| p12 | *Tb*AdoMetDCΔ26 (forward cloning for monomer and heterodimer) | 5'‑AGAT*GGATCC*TTTGAAGGCCCGGAAAAAC‑3' ^a,c^ |

^a^ sequence complementary to an ORF is underlined

^b^ sequence complementary to a vector and used in the ligation-independent cloning is in bold

^c^ sequence recognized by an endonuclease is shown in italics

^d^ mutated nucleotides are shown in lower case.
