# Supplementary material for: Relief of autoinhibition by conformational switch explains enzyme activation by a catalytically dead paralog
Source: eLife. 2016 Dec 15;5:e20198. doi: 10.7554/eLife.20198 (PMC5201418; doi:10.7554/eLife.20198)
Supplement: Supplementary file 2. — Residue positions are highlighted according to conservation: mainly hydrophobic (yellow), mainly small (gray), invariant residues in AdoMetDC enzymes that likely contribute to catalysis (black), coevolving residues that likely contribute to activation (magenta), conserved trypanosomatid prozyme/enzyme residues that stabilize the prozyme/enzyme interaction (orange), and conserved trypanosomatid prozyme/enzyme residues that form the N-helix pocket (wheat). Sequences are labeled according to NCBI GenInfo Identifier (GI) followed by the species. Alignment sections are labeled above according to taxonomy group. Non-conserved termini and insertions are removed from some sequences, with the numbers of omitted insertion residues that belong to unconserved positions indicated with brackets. Aligned sequences were ordered according to taxonomy, with the exception of protist sequences, which were split according to their similarity to other sequences in the alignment (Sphaeroforma arctica and Salpingoeca rosetta sequences are closer to metazoa than other protists) and to distinguish the Trypanosomatid group that contains prozyme sequences. Trypanosomatids were supplemented with sequences from additional species present in the NR database (Phytomonas sp. isolate EM1, Angomonas deanei, and Strigomonas culicis), and indicated prozyme sequences were extended to the N- or C-termini using TBLASTN (marked by *). The confirmed AdoMetDC domains were found to be fused to several additional domains in select sequences, including an AdoMetDC leader (pfam08132) in 35 plant sequences, an f-box-like domain (pfam12937) in one fungal sequence, pyridoxal-dependent ornithine decarboxylase (pfam02784 and pfam00278) in 9 apicomplexan and 3 plant sequences, and protein prenyltransferase alpha subunit repeat (pfam01239) in one Blastocystis sequence. The proline residue (T. brucei P31) that alters peptide bond isomerization upon activation belongs to the Trypanosomatid AdoMetDC sequence motif "FEGPEK [file elife-20198-supp2.pdf]

```
Activation Residues                                                                            cis/trans isomerization
                                                                                                         *
Trypanosomatids Prozyme:
72390539  Trypanosoma brucei TREU927     -----------MSVTRINQQTECPSSVHDLVSCWGGCTQSKT---STDSGLEKRFELNFAQPV----
71408277  Trypanosoma cruzi CL Brener    ----------MLESTWAAAREEVPESVHALMAMWGGFDGPRN---ANDCGIEKRLELDFRGVI----
157872786 Leishmania major Friedlin*     ----------MPTNSWASSRDVFPESVRALMSLWGGFSNPTS---YSDSGLEKRLEFDFAAAV----
154341969 Leishmania braziliensis*       ----------MPPHSWASPRDVFPESVHALMSLWGGFANPTN---YNDSGLEKRLELDFDTSV----
588319214 Phytomonas_sp._isolate_EM1     ------------MPSWTCSRDEYPQSVHALMSLWGGFAPLTE---VSDSGLEKRLEVDFKFPC----
528258165 Angomonas_deanei               ------------MTSWASPRDVYPSSVHDLMSLWGDFSTAPN----SDNGLEKRFEVDFARHV----
528251074 Strigomonas_culicis*           ------------MTSWASPRDAYPSSVHALMSLWGDFSTAPG----SDSGLEKRFEVDFAQPI----
Trypanosomatids:
72390527  Trypanosoma brucei TREU927     ------------------MSSC-KDSL-SLMAMWGSIARFDPKHERSFEGPEKRLEVIMRVVDG---
71408275  Trypanosoma cruzi CL Brener    ------------------MLSN-KDPL-SLMAMWGSVKGYDPNQGASFEGPEKRLEVIMRIIDE---
157872783 Leishmania major Friedlin      --------------MNVCSNTT-KDPL-TLMAMWGSMKGYNPEQGFSFEGPEKRLEVILRSTLE---
154341967 Leishmania braziliensis        --------------MNVSLNTT-KDPL-TLMAMWGSMKRYNPEQGFSFEGPEKRLEVILRCTLE---
588319215 Phytomonas_sp._isolate_EM1     -------------------MSS-KDPV-TLMAMWGSMKDYNPDQGFSFEGPEKRLEVIIRFLPE---
528270660 Angomonas_deanei               --------------MS-SSSCN-KDPL-TLMAMWGSMRGYNPDQGFSFEGPEKRLEIIVRFLDE---
529494686 Strigomonas_culicis            --------------MSVSSPST-KDPL-TLMAMWGSMRGYNPEQGFSFEGPEKRLEIIVRFLDE---
protists:
831777486 Acytostelium subglobosum LB1   --MPIMTTN-KTFNESDAGF-----------------EVEEQCFPGGFEGPEKKLDIRFHPIN[5]Q
166240588 Dictyostelium discoideum AX4   --MPILST--EIFNEADAGFV---------------YQSNSNDYSDGFEGPEKKLDIRFGPIS[9]S
330840910 Dictyostelium purpureum        --MPIMST--EIFNESDAGFV---------------IESTQNCYADGFEGPEKKLDIRFCPIN[6]S
470246992 Dictyostelium fasciculatum     --MPIMSSQ-KIFNEAEAGFAHCI[5]NNTTTPL[8]QEDQETFPGGFEGPEKKLDIRFCSKY[4]S
290984948 Naegleria gruberi NEG-M        NGKPILVN--YAEKHVKHVDC-------------------ECCAEAGFEGPEKRLELDFRPIA[22]
676381665 Aureococcus anophagefferens    -----------------------------------MDESSVPESVGVFEGPEKTLEVCFLPGV----
813169519 Saprolegnia parasitica         IDQAIPDSTNNVSSDTASSTDS------RGDQPV-------MDYPNTFEGPEKNLEVDLVPACD--G
673049047 Aphanomyces invadans           -MPTTDDMKTTAASATA-STDSCS---NCSDTPA------TVDYPNTFEGPEKNLEIDFTPA-----
675188663 Phytophthora parasitica        MAQPFPEPIKLHDDETS-STQS-----EASDT-----------VPNTFEGPEKNLEIDFKLGV----
294883519 Perkinsus marinus              -MYDFPTQGPPGMASDIKQLMREERRDSQEDS-----------QEPTFEGTEKRIEIDFVWSGE---
551667574 Guillardia theta               -----------------------------------MPEIIETNQLFCFEGSEKKMEIDFRPID[6]I
fungi:
629651635 Eutypa lata                    ------MVNLAIPSDYTFSPSE--GTPHLTINHDVA--ADLD-STNAFEGPEKLLEVWFAPGP[5]S
261202444 Blastomyces gilchristii        ------MVNIEILKNYTASPSSFMGTSSLTINHEAT--LDLD-SSTAFEGPEKLLEVWFGPSP[5]S
50553544  Yarrowia lipolytica            ------------------------MILEHHVNHQVA--VDLD-SAEAFEGPEKLLEVWFAPNQESLE
891574827 Schizosaccharomyces cryophilus ------------------------MSPILVVDQENA--QEFN-SS-SFEGPEKLLELWFSAPF[6]G
6324521   Saccharomyces cerevisiae       -----------------MTVTIKELTNHNYIDHELS--ATLD-STDAFEGPEKLLEIWFFPHKKSI-
444315950 Tetrapisispora blattae         -----------------MSASTIPQETTSFIDYEKS--NNLD-STDAFEGPEKLLEIWFYPET[6]A
149237635 Lodderomyces elongisporus      ------M----------VSPPAY--HQDSFVDHELS--KNLD-STYAFEGPEKLLELWFWKSE[5]C
953449703 Mixia osmundae                 GHSAHTNLPLARQHAKHDDHFDPPHHENKANDSGVE--ILQDSAPGFFEGPEKLLELWFAPSE[25]
331247566 Puccinia graminis              -----------------------MVTCVLTKDSTSG--AEPA-NGPPFEGPEKLLELWFAPSA[19]
955498480 Moesziomyces antarcticus       QRIKSLEIMDARAELLRSKFQDDASSIITAVDSNSG[8]FHD-PCGPFEGPEKLLELWFAEEP[14]
914974225 Tilletiaria anomala            --------------------MTATTSSQLKSTKEQE--DWME-AQGPFEGPEKLLELWFAEGP[22]
animals:
74275346  Homo sapiens                   ----------------------------------------MEA-AHFFEGTEKLLEVWFSRQQPDAN
936678339 Trichogramma pretiosum         --MMSMEEEGNKKKLEQQQQGSDSDVEAGA----------EIMS-VQFFEGVEKLLEIWFSTNV[21]
157115295 Aedes aegypti                  --MAE-----NEDLSNSS--SSSSSSR--------------ED-MHFFEGVEKLLEIWFE---PNPS
24583360  Drosophila melanogaster        --MLE------------------------------------NG-SHFFEGVEKLLEIWFEESS----
910327857 Papilio xuthus                 --MAE---------------TEIISNSE-------------SN-DQFFEGVEKLIEIWFT------P
871243907 Aplysia californica            CIMAS----------------------------------AA-SNECFFEGTEKLLEVWFSSSH---P
675851988 Helobdella robusta             --MKN---IE-----------------PGSHCTISVVEDPLHEGEYFFEGTEKLLEIWFSPSS[8]Q
156370797 Nematostella vectensis         --MCE---------------------------------------VGFFEGPEKLLEIWWRPTL[15]
761906225 Amphimedon queenslandica       --MED-------------TNADLSPRPAEPHPAADLSRRFSIEEVGFFEGTEKLLEVWFD--IPSPE
198416848 Ciona intestinalis             --MSV---VD---------VKDGAP------------------QSWFFEGVEKLMEIWFSSSA--PD
268561468 Caenorhabditis briggsae        --MSA-------------TATICS-EKNFAVQTHPV---KAPDEEYFFEGAEKLLELWFCSTK---G
312078795 Loa loa                        --MST-------------STLSVGSLDEITFSVEDV---PSVIEDYFFEGAEKLLEIWFDKNQ----
protists (seq. close to metazoa):
929755270 Sphaeroforma arctica JP610     --MGN-------------DNTAKDVVVEGVV-------------SDFFEGAEKLLELWFE-----PS
514686471 Salpingoeca rosetta            --MDN---------------------------------------NDFFEGQEKLLEVWFKPPN[11]
plants:
985701157 Solanum tuberosum              ------------------------------------MEMDLPVSAIGFEGFEKRLEISFVEPGLFAD
731352674 Beta vulgaris subsp vulgaris   ------------------------------KNDNETGQ---PVSAIGFEGFEKRLEITFSNVPMFSD
302803606 Selaginella moellendorffii     ------------------------------------------MPTPGFEGFEKRLEVEFFPAA----
168048070 Physcomitrella patens          ---------MGATACV-------------SEVAPVCGA---LGTSPGFEGFEKRLEIEFQPAPVFRD
702445187 Eucalyptus grandis             ------------------------------------------MAASGFEGFEKRLELHFTGDE--PA
923724061 Brassica napus                 LSPHTNTNTHF--LCSVLVATSSVLFYPQ----FFS--DL-PMAVSGFEGFEKRLELRFFNDS-ITN
514802427 Setaria italica                GEKKFQSAAYS--NC---------AKKPS-----YA----HPVSAIGFEGYEKRLEITFSEAP[3]D
514756085 Setaria italica                ------------------------------------------MASSGFEGFEKRLELVFSLPS[4]A
926775472 Monoraphidium neglectum        -------------------------------MADR-R-PSMVLPNPIFEGSEKRVEIDFAPSA---S
159470359 Chlamydomonas reinhardtii      --------------------------------MTV-N-ENGVLHMPIFEGSEKRIEVDFRLGP---N
308810503 Ostreococcus tauri             ------------------------------------TMVDGLYPSPHFEGAEKRIEIDFHLNK----
612398587 Bathycoccus prasinos           -------------------------------------MQSELYPSPHFEGVEKRIEINFRVAA[9]I
```

Activation Residues

position anchor strand loop
*

**Trypanosomatids Prozyme:**		
72390539	*Trypanosoma brucei TREU927*	----DIGTVTVKQLASVMERAGESLRQNSAELGIHTLKFDRSLLVFTAKQIVVRSSVSVMLHEAVHP
71408277	*Trypanosoma cruzi CL Brener*	----VPSEVAVLDLESVMSHAGELLERHSAEKGLISLVFGNSLMQLTQRHVIVTSSSSVLLHEILGP
157872786	*Leishmania major Friedlin**	----DVQTFAVEELEDVLAAAGQKLQHHFSAEGLLSLEMTQCIIILTPCKLVVTSASEVMLHQVITP
154341969	*Leishmania braziliensis**	----DVQTFKVAELEDVLAAAGQELQHHSPAKDLLSLEMTQSILIVMKQKLVVTSASEVMLHQLITP
588319214	*Phytomonas_sp._isolate_EM1*	----PIGKLTVADIEALMRNVDEYLENYSCEGDFQSFTFSQTLIVLTSKMLMMSSNSTVMLHHVLKP
528258165	*Angomonas_deanei*	----GQDELCTADIEIILAAADQCLLEHSCGGAMQSLTFSEALVLFNGRQFIVTADSSVHLHKLVSP
528251074	*Strigomonas_culicis**	----TAATFTVAEMEEVFAAADQYLVSQKCEYQVTALTFAQVLVALGSQQMVASADSIVQLHKVIAP
**Trypanosomatids:**		
72390527	*Trypanosoma brucei TREU927*	THVSGLLAHDDDVWQKVIDAICAHIVSREFNEYIRSYVLSESSLFVMKDRVILITCGTITLLNCVPL
71408275	*Trypanosoma cruzi CL Brener*	THSEGLHALGDEVWKGVVGSLNAQIVSKESNEYIRSYVLTESSLFVMRDRIILITCGTTTLLNAVPF
157872783	*Leishmania major Friedlin*	THVDGLRSLDDSVWSGVVGSLNAQIVSRESNEYINSYVLTESSLFVMKNRIILITCGTTTLLNSIPN
154341967	*Leishmania braziliensis*	THVDGLRSLDDSVWTRVVGSLNAQIVSKEANEYINSYVLTESSLFVMKSRIILITCGTTTLLNSIPN
588319215	*Phytomonas_sp._isolate_EM1*	THPDGLYSLEDDVWARVVGTLNAQIVSKENNAHIKSYVLTESSLFVMRNRIILITCGTTTLLNSIPE
528270660	*Angomonas_deanei*	TNKEGFLSFEDSSWEHVVGSLNAEIVSREKNGQINSYVLTESSLFVLRNKIVLITCGTTTLLNSVPI
529494686	*Strigomonas_culicis*	TNKDGFFSFEDAAWEKVVGALNAQIVSRESNEFIKSYVLTESSLFVMKNKIILITCGTTTLLHSVPD
**protists:**		
831777486	*Acytostelium subglobosum LB1*	PSRLGLRTLNKERWQSVLDSARCTIISQTSNDFMDSYVLSESSLFVYPRRVMVKTCGTTTLLHLVDK
166240588	*Dictyostelium discoideum AX4*	PSKVGLRTIDKEKWQTVLDSARCTIISQTSNDHMDSYVLSESSLFVYPRRAMIKTCGTTTLLHLIAK
330840910	*Dictyostelium purpureum*	TSKIGLRAINKDRWQQVLDSARCTIISQTSNQFMDSYVLSESSLFVYPRRAMVKTCGTTTLLHLIAK
470246992	*Dictyostelium fasciculatum*	GPKSGLRSIGKEQWQSVLDSARCTIISSTSNQYLDSYVLSESSLFVYPRRVMIKTCGTTTLLHLIEK
290984948	*Naegleria gruberi NEG-M*	SNFYNLMNVTDETWQSMLDLACCRILSKKTNEHFVSYVLSESSLFVFKRKLVIKTCGTTTLLGILKK
676381665	*Aureococcus anophagefferens*	GRERGCRDLTRAQLDTMLTEARCTILSHISNAHLDAYVLSESSLFVYPFKVVIKTCGRTTLLRCVRP
813169519	*Saprolegnia parasitica*	ADPRGLRALSRAQIDALLDAAKCTILSHVSNAHLDAYVLSESSLFVYPRKMLIKTCGTTTLLKALPL
673049047	*Aphanomyces invadans*	GHPDGLRALSRTQLDALCDVAKCTILSKISNASLDAYVLSESSLFVYPRKLLMKTCGTTTLLKTLPL
675188663	*Phytophthora parasitica*	GHERGLREVPRNDLDEMLEAGQCQILSRTSNEHIDAYVLSESSMFIYAHKIVLKTCGTTTLLKCLPY
294883519	*Perkinsus marinus*	KADLGARAISRSMWDKILALCECTIVSHKALKRLDAYILSESSLFVCADKIIIKTCGTTLLLQGLRT
551667574	*Guillardia theta*	DINGGLRQYNREFWSDIVAILNGTILLADPQEKFDAYLISESSLFVYNDRVIILTCGTTLLLKTLPA
**fungi:**		
629651635	*Eutypa lata*	AKPNGLKAVPSDTWVDMLDMVNCKILSVLDSEHVDAYLLSESSMFVFPHKLILKTCGTTTLLLGLHR
261202444	*Blastomyces gilchristii*	TEPTGLKVVPVEVWKDMLDLVSCKVLSIIESDDVDAYLLSESSMFVFPHKLILKTCGTTTLLSGLPR
50553544	*Yarrowia lipolytica*	GTNKSLRNVSTAQWEQLLNIVQCKVLSSVHTSVLDAFVLSESSMFVFPHKLVLKTCGTTTLLVGIPK
891574827	*Schizosaccharomyces cryophilus*	NTKFGLKNVSRDLWDDMLSRAQCKVLSVVSSNEVDAYLLSESSMFVFPHKIILKTCGTTTLLASLPR
6324521	*Saccharomyces cerevisiae*	TTEKTLRNIGMDRWIEILKLVKCEVLSMKKTKELDAFLLSESSLFVFDHKLTMKTCGTTTTLFCLEK
444315950	*Tetrapisispora blattae*	GENKTLRDIAYEDWVSILKLVNCEILSFKKTSQLDAYLLSESSFFVYDYKVVLKTCGTTTTLFCLQG
149237635	*Lodderomyces elongisporus*	SPRDGLRSIKMDAWAKMLDLVNCKILSMKSSPFMDAYLLSESSLFVFPHKLILKTCGTTTTLACLDE
953449703	*Mixia osmundae*	NALLGLRTVDRPSWERMLDEVHCKVLSVIEGEQVDAYMLSESSMFVWPHKIILKTCGTTTLLLGLNT
331247566	*Puccinia graminis*	PKFTGLRTVNHPLWHAMLDQVKCKVLSVIESEHVDAFLLSESSMFVWPHKIILKTCGTTGLLLGLDS
955498480	*Moesziomyces antarcticus*	PGYFGLRTVPKAVWETMLDVVKCKVLSVIESSHVDAYLLSESSMFVFPHKLILKTCGTTTLLLGLEK
914974225	*Tilletiaria anomala*	LRRLGLKGVPREQWEEMLDIVKCKVLSVIESDEVDAYLLSESSMFVFPHKLILKTCGTTTLLLGLER
**animals:**		
74275346	*Homo sapiens*	QGSGDLRTIPRSEWDILLKDVQCSIISVTKTDKQEAYVLSESSMFVSKRRFILKTCGTTLLLKALVP
936678339	*Trichogramma pretiosum*	ESKHDLRIIPRQKWESLLKIVRCEIISFCRNDSVDAYVLSESSMFVSKRRLILKTCGTTTPLQCLEP
157115295	*Aedes aegypti*	NKNADLRKIPRAMWDALLKTVRCEIISFTRNEQIDAYVLSESSMFVSKRRWILKTCGTTTPLQCLEP
24583360	*Drosophila melanogaster*	NGDDDLRNISRSDWENVLSNVNCQIISTSKNDIIDAFVLSESSMFVSKRRWILKTCGTTTPLKCLGQ
910327857	*Papilio xuthus*	AKQADLRKITRQQWEKVLKIVRCEIISFTKSEQVDAYVLSESSMFVSRRRWILKTCGTTTPLRCVRA
871243907	*Aplysia californica*	NDSCDLRRIERKEWDDLLKLVKCEVISFMRDDSMDAYVLSESSMFVTKDRFILKTCGTTTLLMAVDR
675851988	*Helobdella robusta*	GGRASLRDINRSEWELLLKNVNCEILSSRHSTTMDAYLLSESSMFVSKDRIILKTCGKTTLLEAVKP
156370797	*Nematostella vectensis*	AKKGDLREIPRQKWEKILQLVNCQIISEKRNEDMISYLLSESSMFISKERIILKTCGTTTLLYSIKP
761906225	*Amphimedon queenslandica*	SESRGLRDIPMSFWQVLLDLAKCHVLSKCNNEKLDAYVLSESSMFISRNRFILKTCGTTSLLSTVKP
198416848	*Ciona intestinalis*	GPKKGANLIERWKWENILTHVHCKILDEMKHQDQTAYILSESSLFVSRERIILKTCGTTLCLQALPF
268561468	*Caenorhabditis briggsae*	ITDTSLRSIPRSEIDNMLDVARCKILHSKHNESIDSYVLSESSLFVSDNRVILKTCGTTRLLAALPV
312078795	*Loa loa*	GGATSLRSIPYSELVAMLDIARCHILHSRSSECMDSYVLSESSMFVSDFRIILKTCGTTRLLHTVRR
**protists:**		
929755270	*Sphaeroforma arctica JP610*	ETGQSLRLIKREDIDEFLRVAGCCVVAQCSNEKWDSYVLSESSLFISDDMFLLKTCGTTTLLAAIPN
514686471	*Salpingoeca rosetta*	DDAQGLRAIPRARVEGILDEAKCCILDAFHGDFMDSYVLSESSLFITAYRIILKTCGTTRLLPALPL
**plants:**		
985701157	*Solanum tuberosum*	PNGKGLRSLSKAQLDEILGPAECTIVDNLSNDYVDSYVLSESSLFVYSYKIIIKTCGTTKLLLAIPP
731352674	*Beta vulgaris subsp Vulgaris*	PTGLGLRSLTRAQLDSFLDEAACTIVAELSNSEFDSYVLSESSLFVYPHKIILKTCGTTKLLLAIEP
302803606	*Selaginella moellendorffii*	--ARSLRSLSRDQIDSFLNVAECTIVSQLSNGHFDSYVLSESSLFIYPHRIVIKTCGTTRLLKALPK
168048070	*Physcomitrella patens*	PNGRGLRDLTRSELDSMLCVAECTIVAQLSNAEVDSYVLSESSLFVYPQRIIIKTCGTTKLLCIVPV
702445187	*Eucalyptus grandis*	AVRMGLRRIDFESLERVLHEVQCTVVSAVGNAHLDAYVLSESSLFVYPTKIVIKTCGTTQLLKSVRP
923724061	*Brassica napus*	TNPIGLRLIDFESLDQVLNEVQCTVVSAVANHSFDAYVLSESSLFVYPTKIIIKTCGTTQLLKSIRP
514802427	*Setaria italica*	PHGRGLRALSRAQIDSVLDLARCTIVSELSNKDFDSYVLSESSLFIYPLKIVIKTCGTTKLLLTIPR
514756085	*Setaria italica*	RAQHGLRLLPVGALREALDTVQCAVVSAAGNAAFDAYVLSESSLFVYPSRAILKTCGTTRLLRAVPI
926775472	*Monoraphidium neglectum*	TPAAGLRALSRAQLDELMSLAACTIVSSRTNDHLDAYVLSESSLFVYPTKWVLKTCGTTKLLNAVPR
159470359	*Chlamydomonas reinhardtii*	VPANGLRSIPRETLDELMTLARCCIVSSRSNDKLDAYVLSESSLFVYPTKWILKTCGTTRLLNSVPR
308810503	*Ostreococcus tauri*	ENPRGLREITRTQLDECMTAARCEIVSVRTNVKFDAYVLSESSLFVFPTKLVLKTCGTTHLLAGVPA
612398587	*Bathycoccus prasinos*	PVAGGLRCLSRAALDVCMTAAECEIVSVRSNEKFDAYVLSESSLFVFADKIVLKTCGTTKLLSAVDP

```
                                                              positions anchor strand
Activation Residues                                       displaces N132; forms N-helix pocket   prozyme/enzyme interaction
                                                                Putrescine * *      *                            *
Trypanosomatids Prozyme:
72390539   Trypanosoma brucei TREU927          MLELMRSH----NIIVDWASFMRVNYGSPWDMTSET---MAHEYAELKSAFP-----TGHPYLAGP-
71408277   Trypanosoma cruzi CL Brener         LLDLLRSH----GIDIEWASFMRMNTTSPWSSLCEM---MAQEYAQLKSVFP-----SGHPYLTGP-
157872786 Leishmania major Friedlin*          TIALLTSK----GVRVEWASYLRKNTTSPWCAESEM---MAQEYAELKAAFP-----AGKSFLTGP-
154341969 Leishmania braziliensis*            IIAFLKSK----GVRVEWASYLRKNTTSPWCTDSET---MAQEYAELKAAFP-----TGKSFLTGP-
588319214 Phytomonas_sp._isolate_EM1          LLDLLNQR----SVLVEWMSFSRRNTTAPWSTQCETSDIMAQEYAELKAAFP-----TGQSFLLGP-
528258165 Angomonas_deanei                    LVKMLSEK----NIDIEWASFCRKNISSPWDHN-DTSDVMACEYAELKAAFA-----DGQSFLTGP-
528251074 Strigomonas_culicis*                FMALCARK----GVQAEWASFFRKNTHSPWEHS-DTSDMMACEYAELKSSLP-----QGHSFLTGP-
Trypanosomatids:
72390527   Trypanosoma brucei TREU927          ICEAVSTV----CGEVEWVSFMHKNYSFPWEQKGPH---MAEEFKTLRSHFP-----SGQPFIFGP-
71408275   Trypanosoma cruzi CL Brener         VLDAVSDV----RGEVEWVSFMHKNYSFPWEQKGPH---MAEEFNTLRTYFP-----SGKPFIFGP-
157872783 Leishmania major Friedlin           ILEAISAV----RGELEWVSFMHKNYSFPWMQKGPH---LADEFATLKQHFP-----TGKPYIFGP-
154341967 Leishmania braziliensis             ILEAISAV----RGELEWVSFMHKNYSFPWEQKEPH---LADECATLKQHFP-----TGKPYIFGP-
588319215 Phytomonas_sp._isolate_EM1          ILKAISAV----RAELEWASFMHKNYSFPWEQRGPHTS-LSGEIATLKSHFP-----TGKTYILGP-
528270660 Angomonas_deanei                    IMELISCM----RGEVEWVSFMHKNYSFPWEQKGPHTS-LSEEFNHLKTHFP-----TGKPYILGP-
529494686 Strigomonas_culicis                 ILELISCM----RAELEWVSFMHKNYSFPWEQKGPHTS-LSEEFNHLKAHFP-----TGRPYILGP-
protists:
831777486 Acytostelium subglobosum LB1        MVEVGRE----CGLEIEMVLFSRKNLNNPHKQVFPH---FDDEVKYLNKTFN------GQAYVMGP-
166240588 Dictyostelium discoideum AX4        MVQVGKE----CGLEVEMVVFSRKNLNQPSKQVFPH---FSDEVNFLNKIFD------GQAYVMGD-
330840910 Dictyostelium purpureum             MVEVGKE----CGLEVEMVVFSRKNLNQPTKQIFPH---FNDEVAFLNKIFK------GEAYVMGD-
470246992 Dictyostelium fasciculatum          MTQLAKDT---CGLEVEMLVFSRKNLNNPGKQIFPH---FSDEVDYLNKIFNN----AGHAYVMGP-
290984948 Naegleria gruberi NEG-M             LKEIAAT----QGLEAELVIFSRKNYVFPHRQPHYH---FEEELKYLTDDFP-----GGEGHVVGS-
676381665 Aureococcus anophagefferens         LVDAATKA[5]LGLKLEWVGYSRKNYAFPDEQPSPH---FGEELAYLRAHRD--------GYVLGP-
813169519 Saprolegnia parasitica              LTEYVRD----LGLAVEKVRYSRKNFLLPDDQLFPH---FASEVDYLNDFFP-----NGAAHILGP-
673049047 Aphanomyces invadans                IMEYLQE----LNMAIEKVRYSRKNFLQPEEQHFPH---FSVEVEYLNDFFP-----NGVAHVLGP-
675188663 Phytophthora parasitica             IKTFSER----LGLEIEWVNYSRKNYTFPEEQKYPH---FQTEVAFLSSYFP-----DGSAHVLGP-
294883519 Perkinsus marinus                   LLDHAVNE---LGLELEWLFYSRKSFLFPEFQRGVHG-SLEDEVSVLREVCKDFGCSTGNAYVLGP-
551667574 Guillardia theta                    IMKAGAD----VGLEVCWFQYSRKNFLFPEQQSFPHT-SFDQEVHFLHEYFP-----TGRPFIMGP-
fungi:
629651635 Eutypa lata                         LLRIAAEH[16]AATPWRVFYSRKNFLFPHKQEGPHR-SWKQEVKYLDDMFE-----GGSAYMVGK-
261202444 Blastomyces gilchristii             ILEIAAVY[16]AAAPYRVFYSRKNFLFPDRQRGPHR-SWRDEVKAMDQLFL-----GGSAYMIGK-
50553544   Yarrowia lipolytica                 MLEIARTI[4]ANATPYRVFYSHKTFNCPEKQAPPHK-SWKDEVSYLDSQFD-----NGKAYLVGD-
891574827 Schizosaccharomyces cryophilus      LLEIASS---VGFTHPLRTFYSRKNFLYPERQLAPHT-SWNDEVRYLQLFFP-----KGSSYVVGP-
6324521    Saccharomyces cerevisiae            LFQIVEQE[11]KYKPFKVFYSRRCFLFPCKQAAIHQ-NWADEVDYLNKFFD-----NGKSYSVGR-
444315950 Tetrapisispora blattae              LFNLLFRK[14]KYTPYKVFYSRRCFIFPSKQKSIHQ-KWSDEVKYLDQFFA-----NGRNYLVGR-
149237635 Lodderomyces elongisporus           LFQMTRQICQLTSKEIYRVFYSRRSFMFPEKQIHVHK-DWKHEVSLLNQHFM-----LGKSYVVGN-
953449703 Mixia osmundae                      LLRIAAEH--CGLGKVWRCFYSRKTFMFPERQLGPHR-DWNQEVRFLDGVFD-----NGSAYAVGR-
331247566 Puccinia graminis                   LLKIAKEE--CGFSGAWRWFYSRKAFMFPAQQSAPHT-SWEDETRFLDHRFA----PHATAQVLG--
955498480 Moesziomyces antarcticus            LLTIATRA[50]GAVVKHAFYSRKSFMFPERQKGPHR-DWMLEVAVLDRFFD-----YGSAYTVGK-
914974225 Tilletiaria anomala                 LLEIAFSA[34]GSLVSRCFYSRKSFMFPERQKGPHR-DWMTEVATLDAFFT-----NGSAYTVGK-
animals:
74275346   Homo sapiens                        LLKLARDYS--GFDSIQSFFYSRKNFMKPSHQGYPH---FQEEIEFLNAIFP-----NGAAYCMGR-
936678339 Trichogramma pretiosum              LLDLVESYT--GFDKVENLFYSRKNYKRPELQIAPH---FEEEVTLLDTFFP-----DGEAYCLGS-
157115295 Aedes aegypti                       LLRLASEIA--GYGEIEDLFYSRKNFKRPEMQVSPH---FEEEVAFLDSFFE-----DGRAYSLGA-
24583360   Drosophila melanogaster             LLKLAEAN---GYNVVADLFYSRKNFTRPEAQITPH---FTEEVTYLDSIFP-----NGRSYCLGS-
910327857 Papilio xuthus                      VLALARDTA--GHARVHNVFYSRREFARPHAQLTPH---FDSEVELLDSFFG-----DGRAYIMGP-
871243907 Aplysia californica                 LVELVKQRC--GYDTVADIFYSRKNFTRPELQHHLH---FDKEVEHLDNMFK-----NGAAYVLGR-
675851988 Helobdella robusta                  LLGLAKEQC--GFDNIVDVFYSHKNYLRPDLQPETY---FDQEVAMLDTLFE-----DGAAYVLGK-
156370797 Nematostella vectensis              LLNLAAEEC--GLTIVQDLFYSRMNYLKPDMQKDLH---FEKEVQWLDKLFA-----NGAAYSLGR-
761906225 Amphimedon queenslandica            LLKTVKKFY--PRSEVKDFFYSHSPFMKPDLQLHPH---FNDESTYLDTIFP-----DGAAYVLGR-
198416848 Ciona intestinalis                  IIQLVQDEC--GFDTVADFFYSRKNFIKPDNQPHPY---FQDETVYLDTILS-----ESASYCLGR-
268561468 Caenorhabditis briggsae             LMQLAGTYA--GLDQVQSVYYSRKNFLRPDLQPNLH---FDAEVEYLDSFFE-----DGHAYCLGS-
312078795 Loa loa                             ILHLAKIYC--NVDNVVSVFYSRKSFIRPERQPFPH---FETEVDYLEEHFT-----GGSAYCIGP-
protists:
929755270 Sphaeroforma arctica JP610          MISLAKDL---GRNNVDDVFYSRNQFMEPEQQPAPH---FEEEVKFLDSLF------DGAAYQLGR-
514686471 Salpingoeca rosetta                 IVQAAADV---GLTEIDDMFFSRREFIAPHTQEFPH---FREEVAYLDQYFD------GVAYPLGR-
plants:
985701157 Solanum tuberosum                   ILRLAET----LSLKVQDVRYTRGSFIFPGAQSFPHRH-FSEEVAVLDGYFGKL-AAGSKAVIMGSP
731352674 Beta vulgaris subsp vulgaris        VLKLADS----VGLSVLSVLYSRGTFIFPNAQVAPH---FSEEVSYLNRYFGNL-GSGPHAYVLGDP
302803606 Selaginella moellendorffii          LLAVAASIG--LARVVR-CKYTRGSFLFPEVQPFPH---FDEEVRTLDEHFGSL--HSSKHVVMGD-
168048070 Physcomitrella patens               LLEVVAR----LAMKVRRVKYTRGTFMFPAVQPYPH---FSEEVGYLEKYFGGI-GSGGRAYVMGNR
702445187 Eucalyptus grandis                  LVAHARD----LGLTLCLCRYTRGSFIFPLAQPFPH---FPEEVAYLEGCLPTN-VCSRKASIMRSH
923724061 Brassica napus                      LIHLARN----VNLTLRACRYSRGSFIFPNSQPFPY---FEDEVVIVEESLPKS-LRYRKASVMTPS
514802427 Setaria italica                     IIELAEE----LSMPLAAVKYSRGMFIFPGAQPAPH---FSEEVAALNRYFGGL-KSGGNAYVIGDP
514756085 Setaria italica                     LLRAAAGE---LGLALRSCRYSRGSYLFPEAQPFPH---FADEIRFLDGAVLSA-LRFRRSSVMPPS
926775472 Monoraphidium neglectum             LLELASQG---LGMAPRRCKYSRASFLFPEQQPAPY---FDQEARFLRTHFGHL-GNGGSAYVLGDM
159470359 Chlamydomonas reinhardtii           LLEVARS----IGLEPRRCKYTRASFLFPENQPFPY---FEHEVGFLDEHFGAMCANGGTAFMLGEP
308810503 Ostreococcus tauri                  ILKYAKE----VGMEPRRVKFTRSSFDKPDLQPALH---FDDEVDFLEKHFGGLSIEGGSSYILGSK
612398587 Bathycoccus prasinos                ILERAASIER-LKLVPSSVRFTRSTYLFPEEQREHH---FTDECEFLEGRFGHL--GSNSSYVMGSN
```

```
                                                     shift+3;prozyme/enzyme interaction
Activation Residues                                       shift+1
                                                        *   *
Trypanosomatids Prozyme:
72390539   Trypanosoma brucei TREU927     --VDRDHCFYFVYDGIDRDPS-----SCRRENDVQINVYMYNVQADDEY-DLDG-------------
71408277   Trypanosoma cruzi CL Brener    --LDSDHFFYFVYDAIERNVL-----GAPPEDDVQINVYMYNVKTDGSG-DDDD-------------
157872786 Leishmania major Friedlin*     --VDGHHCFNFVYDNVERIA-------GRKEEDVQVNVFLNDVAAGLEE------------------
154341969 Leishmania braziliensis*       --VDGHHYFNFVYDGVNRVA-------ERKEEDVQVNMFLYDVAEGLDE------------------
588319214 Phytomonas_sp._isolate_EM1     --VDSEHYFFFTFDAIDRSE------GNAVEQDVQINVFLYDIVRDEDDE-LA--------------
528258165 Angomonas_deanei               --IDGEHFFHFIYDNIQRAAAV---ESGRVEEDVQLNAYFYNIQNDET-------------------
528251074 Strigomonas_culicis*           --IDGDHFFHYIYDNIERGD-----ERARVEVDTQLNLFLHGLKADQV-------------------
Trypanosomatids:
72390527   Trypanosoma brucei TREU927     --IDSDHYFLYFHSDVVQP---------SCSDDAQLSMTMYGLDRNQT-KHWYSDKM----PTGPET
71408275   Trypanosoma cruzi CL Brener    --VDSDHYFLFVYDDVIRPC--------ETENDTQLSMTMYGLDRTQT-KHWFSDRF----STGTET
157872783 Leishmania major Friedlin      --VDSDHYFLFCYDDIIRPC--------SSEDDTQLSMTMYGLDKEQT-KYWFRDRF----STSAET
154341967 Leishmania braziliensis        --VDSDHYFLFCYDDIIRPC--------NSEDDTQLSMTMYGLDKEQT-KYWFSDRS----STNAET
588319215 Phytomonas_sp._isolate_EM1     --VDSDHYFFFCYDDVIRPY--------SNEDDTQLNMTMYGLDKEQT-KYWFSDRF---ISTSAET
528270660 Angomonas_deanei               --VDSDHYFFFCYDDIIREEGN-----TVYEADAQLSMTMYGLDHNQT-KHWFHDKF---ECTNDIT
529494686 Strigomonas_culicis            --VDSDHYFFFCYDDIIRDTSK---LDTGYETDAQMSMTMYGLDPNQT-QYWFNDKF---ECTNEVT
protists:
831777486 Acytostelium subglobosum LB1   --LTKDHWNLYIADMRQGN--------TLQRTEQTFEVMMHDLDQEVM-KQFFKR-------EGVTA
166240588 Dictyostelium discoideum AX4   --VNKDHWNLYIADFRKNP--------TLQRTEQTFEVMMHDLDETVM-KQFFKR-------EGVSA
330840910 Dictyostelium purpureum        --VNKDHWNLYIADYRKNK--------TVQRTEQTFEVMMHDLNPEVM-KQFFKR-------EGVSA
470246992 Dictyostelium fasciculatum     --LNKDHWNCYIIDNRRSS--------TIRRTEQTFEVMMHDLDPQVM-KQFFKR-------DGVSA
290984948 Naegleria gruberi NEG-M        --VLSDHWYIYICDMQGVDDQ------ITKDPDQTLEVLMHDLDPAVM-RQFMEEARK----ERAPG
676381665 Aureococcus anophagefferens    --ITGDHWFVYVADQCERP--------AASATERTINIMMFDLGEDVR-DAFYLKPGE---DLRSAA
813169519 Saprolegnia parasitica         --INADHWFAYAWDVHEHGEPVA--KPSAGVKEHTLHILMQDMHEDAA-AAFFKRD------NGWDG
673049047 Aphanomyces invadans           --ITGDHWYSYNWDADTHGPNS[5]VCDERKSEHTVHMLMQDMHADAA-AHFFKRD------NGMDG
675188663 Phytophthora parasitica        --LNHDHWYIYAWDADIHEQPG[7]LKEPRANESTLQVLMQDMHPSVA-KQFFKNND-----STMTS
294883519 Perkinsus marinus              --LNGDHWIMWNADFKEVD--------SNYRYDHNLDIMMYDLPDDVR-SKFFSSSA-----SGTVA
551667574 Guillardia theta               --LSSDHWYLFVADFIRRETPDG--SRKIVDRDQMLNIYMYGIDPQVA-QYFMKADP[29]EFCATG
fungi:
629651635 Eutypa lata                    --MNGDHWYLYITSPQSPFTPP[26]GGLESNDETLEILMTDLDPENA-KKFYLENA[91]EGHALG
261202444 Blastomyces gilchristii        --MNGEHWYLYLTEPYTVLTPP[33]EEEDEEDETLEILMTDLDEENA-KQFYLDHA[55]EGHGLG
50553544   Yarrowia lipolytica            -TTSDHHWYCYVTGHDDHEEVAAHKDHVISSHDETMEILMTDLLPSRA-QQFFTDRL[49]PGHVLG
891574827 Schizosaccharomyces cryophilus --TNKNHWYLFSDVADESDLFE----SSLDPEDETLEVLMTNMSPERS-LQFYAPSL[23]TGHILG
6324521    Saccharomyces cerevisiae       -NDKSNHWNLYVTETDRSTPKG---KEYIEDDDETFEVLMTELDPECA-SKFVCGPE[12]KGHNLG
444315950 Tetrapisispora blattae         NPSEAGHWNLYITDTNKILENK[6]QTQIDREDETMEMLMTGLDQTQM-KYFHKDIS[5]DDGHEMG
149237635 Lodderomyces elongisporus      -FASDDHWFLYVGGKEDDDCRE[11]FGNKPLDQTFEILMTELDPECA-SKFIYSRQ[9]DLGHELG
953449703 Mixia osmundae                 --INGDHWVLYLTPAMDDALGL[34]SLGMPPDQTLEILMSELDPEAS-RAFFHPIS[7]DDGHAAG
331247566 Puccinia graminis              ----ENPWYLYHRTAPPRSGSA[29]SQLAERDYTLEILMTGLSRRAA-SHFESSEE--LGEGHEAG
955498480 Moesziomyces antarcticus       --MNGDHWLLWMGCEGAREAER[13]PAPCVEDQTLEILMTHLSPASC-ARFVFDEN[27]KGHALG
914974225 Tilletiaria anomala            --MNGDHWLLYMAADQAQGEAG[21]AKQAVQDQTLEILMTSLSPASC-ARYYFADD[25]KGQRLG
animals:
74275346   Homo sapiens                   --MNSDCWYLYTLDFPESRV--------ISQPDQTLEILMSELDPAVM-DQFYMK-------DGVTA
936678339 Trichogramma pretiosum         --EESECWYLYTLNREAPSSTP[21]TNEREPDQTLEIMMTQLDPKVM-SIFTRH-------VCSSA
157115295 Aedes aegypti                  --LNRDCWHLYTLSRGGGGSAN[41]LQLPDPDQTIEILMTDLDPKVM-AVFTKD-------VCNTA
24583360   Drosophila melanogaster        --MNLECWYLYTFSRSDIKITP[6]KNVDSDPDQTIEILMQDLDPETM-SIFYKN-------KFNDA
910327857 Papilio xuthus                 ---EKDCWYLYTLLPLEGTVAA[14]CAGCEPDQTIEILMSDLDPAVM-SIFTRA-------HSASA
871243907 Aplysia californica            --MNKDCWHLYTLEDGN-----------VHQSDQTFELLMWDMCQDKM-KIFTKE-------ISSDG
675851988 Helobdella robusta             --LNKDCWYIYTLDRFN-----------ITEPDQTLEIMMMDVNQDVM-NMFTQS-------YGLTA
156370797 Nematostella vectensis         --VNGDSWYFYTLRQQG-----------VEQPDQTLEILMQDLDPNVM-KAFYTG-------QYANV
761906225 Amphimedon queenslandica       --INGKCWYLYLVESIENP---------LQDPDQTLEILMTEFDSELI-GRFSKL-------SYESA
198416848 Ciona intestinalis             --MNRDCWYLYTLNHPENTSL-----IGVNQPDQTLEILMTNLSVESM-QKFTRCDE----GNVRNA
268561468 Caenorhabditis briggsae        --LKQDRWYLYTFHREVEF-------PANKQPDHTLEILMSDLDEEVL-HKFTKD-------FAVDG
312078795 Loa loa                        --RRQDCWFLYTMVAPRAA---------FPFPEHTLEILMNGLPEDVL-NVFNPS-------VSKDG
protists:
929755270 Sphaeroforma arctica JP610     --LNGDSWYLYMLDHPDG---------SIEKPDQTMEILMMDLDQEAM-KAFHVSP------EYPTT
514686471 Salpingoeca rosetta            --LNGDRWYLYTLNNQLH----------ITCPDVTVEIMMTSLDRTAM-NAFYK--------STLTA
plants:
985701157 Solanum tuberosum              D--KTQKWHVYSASAGS---------VQSNDPVYTLEMCMTGLDREKA-SVFYKT-------EESSA
731352674 Beta vulgaris subsp vulgaris   T-VQNCNWHIYSASCVADVE--------DRRGVVTVEMCMTGLNREKA-AVFYKK-------EGHLA
302803606 Selaginella moellendorffii     ---DRQKWHVYVASESKSDRAGE-AVSKSSNTIYTLEVCMTELDKSVA-RSFFKS------ASIVTG
168048070 Physcomitrella patens          --GKFPNWHIYTACEQNADASG[4]EEEEEECVYTVEMCMTKLGRRSA-GQFWKG------AGRESG
702445187 Eucalyptus grandis             M-AASHSWHVFTACDPDLVMDKG----PAPEDFYTVEMCMTELDRGRA-QKFFMRP--GDGTGDSAG
923724061 Brassica napus                 NSNPSRAWHVFTASADVEP----------DESLVVVEVCMTELDRVNA-RSFFRRK--GDENSDSAG
514802427 Setaria italica                A-RPGQKWHIYYATEYPE------------QPMVNLEMCMTGLDKKKA-SVFFKTS--AD-GHTTCA
514756085 Setaria italica                [5]QQHKWHVYAASTSDDDQHV------DGATHFTVEVCMTELDRTLA-RQFYLTTTPGDGTSHAIG
926775472 Monoraphidium neglectum        --FNGLQWHVYVADAHGADAAYA---PPSGRPFHKFEVCMTDLCRVAA-AAFYRDN-----PAFVSA
159470359 Chlamydomonas reinhardtii      --YQGLQWHVYLADDMLPLGP-------EFKPTYNFEVTMTDLGTEAA-AQFFRDN-----PKFVSA
308810503 Ostreococcus tauri             --LKGVQWHVYVAGDACPVQ--------DARPKASLEVCMTQLNREHCDEHFYRT------DKFVSS
612398587 Bathycoccus prasinos           --FDDAQWHVYVAGDFVEET--------KSEASLSVECCMTSLHREHS-QHFFRENSQEEGVPFVSS
```

```
Activation Residues                                                        position N-terminal helical linker
                                                                                                        *
Trypanosomatids Prozyme:
72390539   Trypanosoma brucei TREU927     ----------------------------NTKEQQLLVSHCA----GEYETLRVSTYGSTHPFASFET
71408277   Trypanosoma cruzi CL Brener    ----------------------------ALKSVQQVVPLSE----TEYEMLRVSTDNTAHPFAAFET
157872786  Leishmania major Friedlin*     ----------------------------VNKTTQRFHALQS----GEYEVMRTFA---GVPCISFET
154341969  Leishmania braziliensis*       ----------------------------VDKTTQRFLALPN----GEYEVTRTFA---GVPCVSFET
588319214  Phytomonas_sp._isolate_EM1     -----------------------PNVGLTRKNTQQLTHLEG----NTYEIMRTFD---DISYISFET
528258165  Angomonas_deanei               ----------------------------NEKDRQSINILPD----GTYTVDKVFP---HAHCATFET
528251074  Strigomonas_culicis*           ------------------------TTPTVKGVNHQLVVLPE----GFYEMHRKFP---EAGSATFET
Trypanosomatids:
72390527   Trypanosoma brucei TREU927     AVIREATGLSEVVD----DSWILHDLQYEPCGYSINAIRG-----SEYQTIHITPE-EHCSFASYET
71408275   Trypanosoma cruzi CL Brener    AAIRKATKLDKVAD----DSWKLHDLQFEPCGYSINTIRG-----AEYQTIHITPE-DHCSFASYET
157872783  Leishmania major Friedlin      AAIRAATHLDRVVD----GTWTLHDLQFEPCGYSINAIRD-----EEYQTMHITPE-DHCSFASYET
154341967  Leishmania braziliensis        AAIRSATHLDRVVD----NSWSLHDLQFEPCGYSINAIRD-----EEYQTIHITPE-DHCSFASYET
588319215  Phytomonas_sp._isolate_EM1     AAIRAATHLDRLVD----ETWTFHDLQFEPCGYSVNALRE-----GEYQTIHITPE-HHCSFASYET
528270660  Angomonas_deanei               QKIRKDTKLDILFN--KEEGWTIHDLQFEPCGYSINAIRG-----SDYATTHITPE-SHCSFASFET
529494686  Strigomonas_culicis            AAIREHTHLCQLFP--SEAGWKLHDLQFEPCGYSINSING-----ANYQTIHITPE-SHCSFASYET
protists:
831777486  Acytostelium subglobosum LB1   WDTTVSSGIANLIP----GSI-IDDFQFEPCGYSMNGLRNK-----AYWTIHITPE-DHCSYVSFDT
166240588  Dictyostelium discoideum AX4   WDTTVNSGIADLLP----GSM-IDDFQFDPCGYSMNGLLNE-----FYWTIHITPE-SHCSYVSFDT
330840910  Dictyostelium purpureum        LDTTVSSGISNLLP----GSI-IDDFQFDPCGYSMNGLLDQ-----WYWTIHITPE-SHCSYVSFDT
470246992  Dictyostelium fasciculatum     WDTTVSSGISNLLP----GSI-IDDYQFDPCGYSMNGMLDK-----WYWTIHITPE-DHCSYVSFDT
290984948  Naegleria gruberi NEG-M        VTIEQQSGIDKLLP----GSE-IDSFEFDPCGYSMNGLLGK-----TYWTIHITPE-DSCSFVSFET
676381665  Aureococcus anophagefferens    RAMTARSGLGALIP----SSSHLDDHAFAPCGYSMNALCFE-----SYTTVHVTPE-ASCSYASFET
813169519  Saprolegnia parasitica         AVMSEKSGIRDLAP----DAI-MDAFAFEPCGYSMNAIDGP-----SYSTIHITPE-SHCSYASYET
673049047  Aphanomyces invadans           AKMSEASGIRDLVP----NAI-LDSFAFEPCGYSMNSIEDD-----VYSTIHITPE-SHCSYASFET
675188663  Phytophthora parasitica        RRMTIESGIRDLVP----GAV-IDDFAFDPCGYSMNGILFD-----AYYTIHITPE-SHCSYVSFET
294883519  Perkinsus marinus              DGMSEESGICDIYP----GAV-VDAINFTPCGYSCNASLDD---GESFFTIHVTPE-ESCSYASFET
551667574  Guillardia theta               DEASDRSGISSLLEHD--GKV-VHSHLFEPCGYSMNGQAAD---GSSYWTIHITPE-AHCSYASFET
fungi:
629651635  Eutypa lata                    TVVSDVCGLSEVYPTSKYPDARIDSYLFSPCGFSANGIVPA[31]THYFTVHVTPE-PVCSFASFET
261202444  Blastomyces gilchristii        TVVAESCGLTDIYPTSKYPGARVDAYLFTPCGFSANGVVPA[17]SHYFTVHVTPE-PHCSYASFET
50553544   Yarrowia lipolytica            AYVSTQSGIADIYPRN-KASVAVDSFCFTPCGYSANAIVDS----GYYTIHVTPE-QHCSYASFET
891574827  Schizosaccharomyces cryophilus TYMAELSGVKELCSTK-DGKSVLDAFQFEPIGFSSNMIYKD-----RYATIHVTPQ-DHCSYASFET
6324521    Saccharomyces cerevisiae       YQMTKNTRLDEIYVN[6]LSFHHDAFAFTPCGYSSNMILAE----KYYTLHVTPE-KGWSYASFES
444315950  Tetrapisispora blattae         NEMTRCSKIDKIYTLDNNSTIISDSFAFTPCGYSSNNIIDE----SNYFTLHVTPE-KGWSYASFES
149237635  Lodderomyces elongisporus      RETMIETELDSVFQP[72]EFLHDAFAFTPCGFSSNSICSN-IGGGYYTLHITPE-SGWSYASFET
953449703  Mixia osmundae                 TRVSRQVGVQDLFP-----DAVLDAFLFEPCGYSANAVLDD-----RYATIHVTPE-QDWSYASFEC
331247566  Puccinia graminis              IQIATSLGIDAPGLLG---SGETDAYLFSPCGFSANLVGGP--DQKRYGTIHVTPE-RSHSFASFES
955498480  Moesziomyces antarcticus       LALSNKLGLTQLLP-----ETEIDAFAFEPCGYSANAVMPR[5]NAGYWTIHVTPE-EDSSFASFET
914974225  Tilletiaria anomala            VALASQLGLTNLFA-----ETHVDAFAFEPCGFSANAVVLN[15]DGYWTIHITPE-EGSSYASFET
animals:
74275346   Homo sapiens                   KDVTRESGIRDLIP----GSV-IDATMFNPCGYSMNGMKSD----GTYWTIHITPE-PEFSYVSFET
936678339  Trichogramma pretiosum         EEATRKSGIDQLLP----NML-IDDFLFEPCGYSMNGVSKN----GSYMTIHITPE-PDFSYVSFES
157115295  Aedes aegypti                  LEATQKSGIHKLIP----GMV-IDDYLFDPCGYSMNGVSKS----GCYMTIHITPE-PDFSYVSFES
24583360   Drosophila melanogaster        NGATVKSGIDTILP----TMH-IDDFLFDPCGYSMNGINDK----GEYMTIHITPE-NQFSYVSFET
910327857  Papilio xuthus                 HHATKESGIDKIIP----GMV-IDDYLFDPCGYSMNGVAKD----GCYMTIHITPE-RSCSYVSFES
871243907  Aplysia californica           KDATQKSGIGTIIP----GLQ-IDDFLFEPCGYSMNGILPG----GFYITIHVTPE-PHCSYVSFES
675851988  Helobdella robusta             SQLTEKTGILDILP----GAI-IDDWLFEPCGYSMNALLPN----GRYFTIHITPE-DEFSYVSFET
156370797  Nematostella vectensis         SEVTKATGIADFIP----GAI-IDDFLFDPCGYSMNGLFGD-----NYFTIHITPQ-KECSYVSFET
761906225  Amphimedon queenslandica       EDLNKSTGLNDLIP----GAT-IDSYIFEPCGFSLNAIVKE----DMYFTIHVTPQ-EECAYVSFET
198416848  Ciona intestinalis             AKFTEDIGISDILP----GSK-INSILFDPCGYSMNGLLDK----NQYWTIHVTLE-PEFSYVSFET
268561468  Caenorhabditis briggsae        KDCFMRAGIDKIIPA---GAD-VHDELFDPCGYSMNAYIND---TDQYATIHVTPE-KEFSFASFET
312078795  Loa loa                        KDCRMKSAINTILPP---GII-VHEELFNPCGYSLNGLIPH---SDHYIMIHVTPE-PDFSYVSFET
protists:
929755270  Sphaeroforma arctica JP610     EVVTKKSGIADFLP----GAM-TSGALFNPCGYSVNGLLGD-----AYFTIHVTPQ-EGFSYASFET
514686471  Salpingoeca rosetta            REVTQQSGIADIVP----GAH-IHEFLFDPCGYSCNAVFGE-----FYFTIHVTPQ-EEFSYVSFET
plants:
985701157  Solanum tuberosum              AHMTVRSGIRKILP----KSE-ICDFEFEPCGYSMNSIEG-----AAVSTIHITPE-DGFTYASFES
731352674  Beta vulgaris subsp vulgaris   KEMTKMSKINDIIS----SHE-ICDVDFDPCGYSMNGVDG-----GAYSTVHVTPE-DGFSYASYEV
302803606  Selaginella moellendorffii     EDMTRASGINSILP----RSQ-ICDFAFDPCGYSMNGLEN-----GAHSTIHITPE-DGFSYASFEC
168048070  Physcomitrella patens          EEMTAGANIQSLLP----ESR-ICDFAFEPCGYSMNGIEG-----AAHSTIHVTPE-EGFSYASFEA
702445187  Eucalyptus grandis             KEMTALTGIGEINP----GAL-ICDFAFDPCGYSMNGIDG-----DRYSTIHVTPE-EGFSYASYEC
923724061  Brassica napus                 KEMTRLSGIDMINA----NAF-ICDFAFDPCGYSMNGVDG-----DRYSTIHVTPE-DGFSYASFEC
514802427  Setaria italica                KEMTKLSGISEIIP----EME-ICDFDFEPCGYSMNAIHG-----SAFSTIHVTPE-DGFSYASYEV
514756085  Setaria italica                DAMTAASGLGDVNP----RSL-AFGYAFAPCGYSMNALDG-----ARYATVHVTPE-DGHSYASYEC
926775472  Monoraphidium neglectum        ARTTTDTGIRGLVP----GAD-IDDYVFEPCGYSMNGILG-----GGFITIHITPE-DGFSYASVEV
159470359  Chlamydomonas reinhardtii      ERTTKDTGIAELKP----KAI-IDDYVFEPCGYSMNGIDN-----TGLITIHVTPE-AHQSYASVEF
308810503  Ostreococcus tauri             AQTTTDSGIRSIFE----EFD-IDDYVFEPCGYSMNGLNEHGSIDGQYSTIHITPE-EGFSYASCEL
612398587  Bathycoccus prasinos           EKTTHDSGIQSIFE----SFS-IDDYVFEPCGYSMNGLNEE--YSGEYSTIHITPE-DGFSYASVEH
```

Activation Residues

```
Trypanosomatids Prozyme:
72390539  Trypanosoma brucei TREU927      N[4]ASDITK---ILLKKFY--PERVLLVLLQDR[13]LEGFTVVHRGANHFGGG-YVFHQATYARS
71408277  Trypanosoma cruzi CL Brener     N[4]ATKKRE---LLLEKFC--PDRFTMVVLQDR[13]MEGYTIMNRATNHFGQG-YAFHQTSYIRA
157872786 Leishmania major Friedlin*      NAKAAVASPT---RLLDTFQ--PTHFTVAALQDR[13]FTPYTLQNRTVNLFGEG-YAFHQLLFARS
154341969 Leishmania braziliensis*        NAKAAVTTPK---RLVDTFR--PAYFTIAALQDK[13]FHAYTLQNRTVNFFGEG-YAFHQLLFARS
588319214 Phytomonas_sp._isolate_EM1      N[6]SPARLE---SLLKKYK--PSKIVMFALQDR[13]LPGYALQSSLTNHFREG-YSFYQLTFVCG
528258165 Angomonas_deanei                N[6]SKTKIA---SLLEQHQ--PARFTIIFFEDK[12]FDSYALNHKSVNYFGMG-YAFHQLVFEKC
528251074 Strigomonas_culicis*            N[6]DTSKIA---ALLAAYE--PTRFTIVCLEDT[12]FEHYEQKRRTANYFGAG-YSYIHSVFERT
Trypanosomatids:
72390527  Trypanosoma brucei TREU927      NTC-ALNYSK---CVLRVFD--PERFSVIVFIDP[20]YPNYEAHHRTVNEYTPG-HWVLKVNYVKR
71408275  Trypanosoma cruzi CL Brener     NTP-AVNYSE---RVLGVFA--PIRFSVIVFIDP[20]YPKYELQNRTVNEFAPG-YVVMKMNYARR
157872783 Leishmania major Friedlin       NSR-ATNYSD---RVLGVFR--PQRFTVIVFLDP[20]YPEYSLLHRTTNEFAPG-YVAMKINYVRT
154341967 Leishmania braziliensis         NSR-AANYSD---RVLGVFR--PQRFTVIVFLDP[20]YPEYRVLHRTTNEFAPG-YVAMKINYIKT
588319215 Phytomonas_sp._isolate_EM1      NCH-DSNYADRIKNVLSVFR--PQRFTVLVFLDP[20]FTEYEVFNRTVNEFTPG-YVAMKINYVRS
528270660 Angomonas_deanei                NAN-EANYFKTVLKVLDVFR--PQRFTVIVIIDP[36]EVDYCVMHRAVNEFAPG-YVAMKINYVQR
529494686 Strigomonas_culicis             NKD-ADNYFATVRNVLRVFR--PQRFTVIVVVDP[40]FIDYCVLHRTVNEFSPG-YAAMKINYIQR
protists:
831777486 Acytostelium subglobosum LB1    NLA-VADYNQ---LVLAVFK--PGRFTAALYCED[14]MPEFAMTNKTIHGFDGG-YDVVVCNFDRQ
166240588 Dictyostelium discoideum AX4    NVA-LADYNQ---LVLNVFK--PGRFTAALYAED[14]VPSYAIQNKTVHGFDGG-YDVVVSNYQQL
330840910 Dictyostelium purpureum         NIA-LPDFNQ---LVLNVFK--PGRFTTAIYSED[15]IPEFSIQNKTLHGFDGG-YDVVVSNFKLS
470246992 Dictyostelium fasciculatum      NIA-LPSYHI---LILKVFR--PGRFTAALYAED[14]LLEHAITNKTVHSFDG[12]FAVERYKDK
290984948 Naegleria gruberi NEG-M         N[5]IENYSE---LVIHTFR--PGRFTVTLFADD[49]GDEYKLKSKTFYEFTGG-YNLTFTNFAKI
676381665 Aureococcus anophagefferens     NTA-LKGYAS---LVLAVFK--PKKVVLTLFADE[22]LGTYHRNDLSLLHVEHD-CVCMMANYSLH
813169519 Saprolegnia parasitica          NDM-VGAYGP---LVVGVFR--PAKFTVTMSVVG[13]FNGYKRRGGWTQAHMDGDITVLKANFAVA
673049047 Aphanomyces invadans            NDK-VGTYAP---MVLAVFR--PRKMTLTMSVLG[13]FKGYKRRGGWTQVHMDGDLISLKANFVIV
675188663 Phytophthora parasitica         NAR-LRSYAS---LVLRCFR--PAKYSVSVYTDR[15]EDMVKIDDDLLYKRKG[12]CRMANYAQT
294883519 Perkinsus marinus               N[7]LVETIR---RAVAVFK--PGRFSLTHVADN[12]DTDYKVKATSTYRWELD-YQASVSSYVSR
551667574 Guillardia theta                N[5]YHHLIA---RVLEVFR--PAKFTTVEHVDA[14]LDGYDLAGRTFNEFGTKDYVVQMCNYVSK
fungi:
629651635 Eutypa lata                     N[10]SEVVE---HVVDIFK--PGRFSITLFEAK[26]IKGYRRIDRIVHDFDD--YELVFRFFERE
261202444 Blastomyces gilchristii         N[10]SEIVE---HVVDIFK--PGRFSVTLFEAK[46]IPGYRRVDRIVHDLDG--YDLVFRYYERN
50553544  Yarrowia lipolytica             N[10]IDVIE---KVLNVFR--PGKLSVTLFEAE[13]LDGYKRTDKVLYEFDG--YQMVYVCFEAI
891574827 Schizosaccharomyces cryophilus N[10]METIE---KAVKTFG--AGNFSLTLFQAK[24]FESYKREEFIVYDFPG--YELVFASFSSV
6324521   Saccharomyces cerevisiae        N[13]LDVLL---HILNVFQ--PREFSMTFFTKN[15]LPDYIKLDKIVYDLDD--YHLFYMKLQKK
444315950 Tetrapisispora blattae          N[12]RNVMQ---RVLDIFR--PQEFCLTLFQNT[19]VPGYVKMDHILYDLDD--YQLLYVRLHRI
149237635 Lodderomyces elongisporus       N[11]LTVLT---KVLKIFR--PRKFSLTLINEI[19]EFGYKKSEKAVYDLKNE-YNLLYLNFVKA
953449703 Mixia osmundae                  N[20]HSLVN---KVLAIFK--PARFSITLFVSR[28]QDAYTRRDRIHHEFDD--YDLGFATYEHP
331247566 Puccinia graminis               N[10]RNVIS---RILKTFE--PKNTTITLFNSH[26]RNLYRLGEQKDHELDD--YNLAYISLQRR
955498480 Moesziomyces antarcticus        N[22]PTLVR---RVVDIFE--PGKMSVTLFVST[34]LKGYKRTDRIAYEFER--YNLVFVSFEKR
914974225 Tilletiaria anomala             N[24]TSLIN---HVISTFE--PGKMSITLFVSN[36]IKGYQRTDRIAYEFER--YDLVFVSFEKV
animals:
74275346  Homo sapiens                    NLS-QTSYDD---LVVEVFK--PGKFVTTLFVNQ[13]IEGFKRLDCQSAMFND--YNFVFTSFAKK
936678339 Trichogramma pretiosum          NVA-AASYGD---VVLRCFK--PGKFVVSIFANK[21]RRDYVRDDAQYCRFKN--YDLSCAFYSKF
157115295 Aedes aegypti                   NVA-SSSYGD---LVIDTFQ--PGKFIVTVFANK[16]IDQWKRRDIQFSRFPT--YDLTYAQYCKY
24583360  Drosophila melanogaster         NVA-LSNYRK---LVINTFK--PGKFIVTIFANK[17]GSHWKRTDMQCCNFPS--YNLLFAQYSHS
910327857 Papilio xuthus                  NVP-VSSYDE---VVLHAFR--PGRFVLTVFATP[16]LCGYEQKEAQYCRFSG--YELHYALYAKF
871243907 Aplysia californica             NVP-Q-SYDD---LVLNVFN--PGKFLMTVFANK[16]IEGYQRMDHQFCSFKN--YFLTYTQFSRP
675851988 Helobdella robusta              NVP-RDSYND---LVTDIFQ--PNKFIVTLMANE[14]VKPFRRTEHQACEMKN--YNLNYALYERP
156370797 Nematostella vectensis          NIR-VKCYKE---LVLKAFK--PGRFLMTLFANE[15]FHGYQRWDLQLCQMKY--YNLTYGHYRQV
761906225 Amphimedon queenslandica        NVV-GECYKS---LVLDIFK--PGSFTLTLFANK[15]LSNYTCLDKQISLFSI--YNLTFLHYKHN
198416848 Ciona intestinalis              NVI-MKDYKE---LVLNLFQ--PQVVSMTLFANH[16]VPGYKRLDRQVAQLQD--YSLWYSQLKKE
268561468 Caenorhabditis briggsae         NQD-LVCLYS---QVLQCFR--PNKILMTVFAND[16]LPGYRRTNVQFVRLET--ETLVYAHFVRK
312078795 Loa loa                         NQN-TLNLSE---QVLEIFRHVPNKFLLTIFTND[16]ICGCHRTNLQFLELPT--ETLVYAQFERA
protists:
929755270 Sphaeroforma arctica JP610      NYL-AEDYTE---LVLKTFC--PGKFVMTVFANP[15]LTGYNRTERQEYQFNK--YNLSYGHFSRD
514686471 Salpingoeca rosetta             DVL-KEDYTP---MVLDIFK--PGKFSINVFANE[17]GAGFQRHDQQLCCLAH--ASVLFGHFTSN
plants:
985701157 Solanum tuberosum               V[8]LGPLVE---RVLACFE--PAEFSVALHADV[12]VKGYSLAEWSPEEFGEG-GSIVYQKFTRT
731352674 Beta vulgaris subsp vulgaris    M[5]SIRFEP---LALRCFE--PKEFTIAVTCFG[14]VKGFTCRSAVKQELPNG-GFVIYKTYVAQ
302803606 Selaginella moellendorffii      M[5]KTGLNA---LVVRCFK--PGFFSVCVHAAP[24]PVAYACSGSHKEELPGG-SAVVYHTFQLA
168048070 Physcomitrella patens           M[5]QVELGA---LVVGVFK--PAAFAMSVDVSG[18]PRGYVCNGSSRQELASG-SAVVFHTFQEA
702445187 Eucalyptus grandis              V[6]GDDIAR---MVAWVFR--PGAMSVSVTSGS[12]PLGLKRRSCATDQFPEA-GTVVFQSFTAR
923724061 Brassica napus                  G[7]QGDIAE---VAIDVFR--PGCVSIATTYNG[17]KLGLKCRSRLMDEFPGS-GTVVYQSFTPR
514802427 Setaria italica                 M[5]ALAYGD---LVLRCFG--PSEFSVAVTIFG[13]AEVYDCNNMVEQELPG--GVLIYQSFSVT
514756085 Setaria italica                 G[3]AGCALA---SAIAVFR--PATVSVSVCYDG[20]PMGLACRSRAAEAFPGA-TTVTYQTFTRT
926775472 Monoraphidium neglectum         T[5]AFCPAD---MIAAIFR--PGSLSASLSVDA[15]PPGYGVQSATAQELATG-GRVSYYTFGPD
159470359 Chlamydomonas reinhardtii       S[6]MAPVQE---LVVKIFA--PGKVSVTMSVDK[15]PAGFGLQGASSQVSPCG-GKVAYYTLIEK
308810503 Ostreococcus tauri              S[5]DLDAFA---YVANVFK--PGKMIFAISVDG[16]IPGYTRVQATRQEFDVE-GVVAYFTYEHN
612398587 Bathycoccus prasinos            S[8]AFSVSN---FCANVFQ--PGKMTFAVTCHG[22]GETYSRPSMCFQQLPKDQGFVAYLTFVKT
```
